# Sun safety education in a maritime climate

**DOI:** 10.1002/ski2.137

**Published:** 2022-05-28

**Authors:** Julie Peconi, Greg Fegan, Rachel Abbott

**Affiliations:** ^1^ Swansea Trials Unit Swansea University Medical School Swansea University Swansea UK; ^2^ Skin Care Cymru Swansea UK; ^3^ Dermatology Department Cardiff and Vale University Health Board Cardiff UK

1

Dear Editor,

With an average 156 days of rain a year in the United Kingdom (UK), it's no surprise that when the sun comes out, the British approach is to ‘make the most of it’. Spend a sunny day at any UK beach and you'll see several sunburns at the end of the day. But summer is fast approaching here, and dermatologists and healthcare professionals continue to stress that skin cancer prevention is of upmost importance.^1^

Skin cancer, including melanoma and non‐melanoma (keratinocyte), is increasing rapidly at a rate of 8% annually and now comprises half of all cancers in England and Wales.^1^ Accounting for an increasing proportion of workload, the disease is impacting dermatology services, causing care for other skin conditions to suffer, and waiting lists to grow.

However, with estimates that 86% of melanomas are preventable with less sun exposure^2^ there is hope. Most skin cancers result from excess ultraviolet (UV) light exposure and severe sunburn as a child greatly increases risk of future skin cancer.^3^ Combine this with the fact that childhood is when individuals start to develop responsibility for health‐related behaviours including attitudes to tanning, teaching children about enjoying the sun safely and skin cancer prevention makes sense.

In line with this, supported by a recent systematic review which showed educational programmes appeared effective for improving sun protection outcomes in children under 18,^4^ major health bodies including World Health Organization, British Association of Dermatologists and National Institute for Health and Care Excellence, all recommend sun safety education and guidance in schools.

Yet while prevention and education are clearly key, in the UK both sun safety knowledge and behaviour need to increase if future skin cancer rates are to decrease.^5^ Additionally, there has been very little research done in UK primary schools^4^ perhaps given the variable weather and the particular challenges in promoting sun safety in a ‘maritime climate’.

In England, it's now a statutory requirement to teach primary school children about safe exposure to the sun. However, in Wales, it's a different story. Despite a strong policy focus on prevention activities, and calls from both the public and charities that schools have clear plans in place, in Wales, it is still not mandatory to teach children how to enjoy the sun safely and there is no blanket guidance to keep children protected.

Informal discussions with several Healthy Schools Co‐ordinators based at Public Health Wales suggest schools in Wales would welcome any additional support in this key area. Unfortunately, while Cancer Research UK and the Wales‐based Tenovus Cancer Care previously offered resources and guidance to schools, both charities have cut their sun safety education and prevention activities due to other priorities. The English‐based charity Skcin has a free sun safe schools accreditation scheme available to schools in Wales, although their resources are not available in Welsh which may pose a deterrent to registration, especially for Welsh speaking schools.

While many will argue that the risks of a child getting sunburnt at school are minimal, the benefits of a sun safety policy in schools reach beyond the classroom. Due to the increased risk of damage from sun exposure at a young age children should be free to enjoy the weather safely, wherever they are. Providing children with a sun safe school environment, educating them on sun protective behaviours and encouraging healthy attitudes towards tanning from an early age are all ways to empower them to take control over their health.

We have recently won funding from Health and Care Research Wales for Sunproofed, a 2‐year scoping study^6^ to better understand the landscape of skin cancer prevention in primary schools in Wales. Bringing together both quantitative and qualitative methods and using anonymised, routine data, we will discover what if any, sun safety policies currently exist, the benefits, barriers and facilitators to teaching sun safety in school and what support schools need in this area. A key output of this research will be to use study findings to co‐produce guidance on best methods for implementing sun safety policies in Welsh schools.

While we acknowledge the long‐term benefits of any intervention that tackles skin cancer prevention will take years to become apparent, we are pleased Welsh Government have recognised the importance of this work. Teaching children how to be safe in the sun has the potential to improve sun protection outcomes,^4^ potentially reversing growing rates of skin cancer and saving both resources and lives. And when the rain stops in Wales? Young people will be well placed to protect themselves.

## AUTHOR CONTRIBUTION

**Julie Peconi:** Conceptualization (lead); Funding acquisition (lead); Project administration (lead); Writing – original draft (lead). **Greg Fegan:** Conceptualization (supporting); Funding acquisition (supporting); Project administration (supporting); Writing – review & editing (equal). **Rachel Abbott:** Conceptualization (supporting); Funding acquisition (supporting); Project administration (supporting); Writing – review & editing (equal).

## CONFLICT OF INTEREST

None to declare.

## ETHICS STATEMENT

Although this letter focuses on our opinion, the study we reference in the letter, Sunproofed has received ethical approval from Swansea University's Medical School Research Ethics Sub‐Committee.

## Data Availability

Not applicable.
